# *Myo*-Inositol Restores Tilapia’s Ability Against Infection by *Aeromonas sobria* in Higher Water Temperature

**DOI:** 10.3389/fimmu.2021.682724

**Published:** 2021-09-10

**Authors:** Man-jun Yang, Ming Jiang, Xuan-xian Peng, Hui Li

**Affiliations:** ^1^State Key Laboratory of Bio-Control, School of Life Sciences and Southern Marine Science and Engineering Guangdong Laboratory (Zhuhai), Guangdong Key Laboratory of Pharmaceutical Functional Genes, Sun Yat-sen University, University City, Guangzhou, China; ^2^Tibet Vocational Technical College, Lhasa, China; ^3^Laboratory for Marine Fisheries Science and Food Production Processes, Qingdao National Laboratory for Marine Science and Technology, Qingdao, China

**Keywords:** water temperature, bacterial infection, *Aeromonas sobria*, myo-inositol, metabolome, innate immunity

## Abstract

Bacterial infection presents severe challenge to tilapia farming, which is largely influenced by water temperature. However, how water temperature determines tilapias’ survival to infection is not well understood. Here, we address this issue from the perspective of metabolic state. Tilapias were more susceptible to *Aeromonas sobria* infection at 33°C than at 18°C, which is associated with differential metabolism of the fish. Compared to the metabolome of tilapia at 18°C, the metabolome at 33°C was characterized with increased an tricarboxylic acid cycle and a reduced level of *myo*-inositol which represent the most impactful pathway and crucial biomarker, respectively. These alterations were accompanied with the elevated transcriptional level of 10 innate immune genes with infection time, where *il-1b*, *il-6*, *il-8*, and *il-10* exhibited a higher expression at 33°C than at 18°C and was attenuated by exogenous *myo*-inositol in both groups. Interestingly, exogenous *myo*-inositol inactivated the elevated TCA cycle *via* inhibiting the enzymatic activity of succinate dehydrogenase and malate dehydrogenase. Thus, tilapias showed a higher survival ability at 33°C. Our study reveals a previously unknown relationship among water temperature, metabolic state, and innate immunity and establishes a novel approach to eliminate bacterial pathogens in tilapia at higher water temperature.

## Introduction

GIFT (genetically improved farmed tilapia, *Oreochromis niloticus*) is one of the most extensively farmed economic fish species in the world due to its high protein content, large size, rapid growth and great adaptability to a wide range of rearing conditions (1, 2). The animal is cultured in more than 100 tropical and subtropical countries, and thereby fishing productivity is improved and the development of aquaculture is facilitated in the world (3, 4). Although tilapia accounts for 7.4% of global production in 2015, the demand for tilapia is increasing in recent years. Therefore, many countries in Asia, Africa, and Latin America expand the tilapia farming for driving the global growth. China is the biggest producer and exporter of the fish, accounting for around 26% of total supply in 2019. In 2020, an increase of 3%–4% compared to 2018 is obtained, which increases income approximately 252 million USD (http://www.fao.org/in-action/globefish/market-reports/tilapia/). Therefore, culture of tilapia is valuable in aquaculture in the world.

However, as tilapia aquaculture has intensified and become more global, disease outbreaks are the main cause of reduced production that results in huge economic loss (5), where bacteriosis continues to be one of the major issues compromising sustainability (6). Among environmental factors that are related to bacterial infection, water temperature plays a crucial role especially due to fish as a poikilothermal animal. The water temperature fluctuates violently as the seasons change (7) and is a major factor in the outbreak of bacterial infection (8–13). Reports have indicated that tilapia is more sensitive to pathogens such as *Streptococcus agalactiae*, *S. iniae*, and *Francisella noatunensis* at higher water temperature than at lower water temperature (8–12). Temperature also impacts vaccine efficacy and innate immune response in cultured tilapia (14–17). Therefore, control of bacterial infection especially at higher water temperature is particularly important for sustainability of tilapia farming. However, how to control the higher water temperature-related bacterial infection is largely unknown.

Like *S. agalactiae* and *Edwardsiella tarda*, *Aeromonas sobria* is one of the most important pathogens in tilapia farms worldwide (18). Higher water temperature (above 30°C) has been designated as a predisposing factor for the disease in tilapias (11). Unfortunately, there are still few effective measures to prevent and control temperature-related infectious diseases in tilapia instead of antibiotics. Thus, development of effective approaches is especially important for tilapia culture sustainability.

Recently, we have developed a functional metabolomics approach to identify crucial biomarkers (19–22). Then, the crucial biomarkers are used to reprogram an antibiotic-resistant metabolome and an infective metabolome to an anti-sensitive metabolome and an anti-infective metabolome, respectively, thereby decreasing the viability of antibiotic-resistant bacteria and elevating the survival ability of hosts infected by pathogens (23–28). We have also demonstrated that the approach is effective in increasing the ability of fish against bacterial infection at higher temperature (11, 13). In the present study, we extended the approach to tilapia infected with *A. sobria* at higher temperature and showed a higher temperature-mediated differential metabolome. Of the selected biomarkers of the differential metabolome, *myo*-inositol was identified as the most crucial biomarker. Myo-inositol is a bioactive compound that lies in dual functions as signals and as key metabolites under stress (29). It modulates innate immunity response and thereby elevates survival of tilapia infected with *A. sobria* at higher temperature.

## Materials and Methods

### Fish and Rearing Conditions

Juvenile GIFT (1 month old, body length, 3–4 cm; body weight, 2 ± 0.2 g) were commercially obtained from a tilapia-breeding corporation in Guangzhou, P.R. China. Microbiological detection confirmed that these animals were free of specific pathogens. Tilapias were cultured as previously described (13). In brief, they were maintained in 25-l open-circuit water tanks with aeration and fed on a balanced commercial diet (Jinfeng^®^, Jiangmen, P.R. China). Water temperature was then slowly changed until these fish were adapted to constant water temperatures at 18°C or 33°C. The water temperatures were monitored regularly. Tilapias were kept at one of the two temperatures for 7 days before all experiments including bacterial challenge, measurement of enzyme activity, and innate immunity genes. The culture and treatment of the experiments were approved by the Institutional Animal Care and Use Committee of Sun Yat-sen University (Approval NO. SYSU-IACUC-2020-B126716).

### Bacterial Strain and Challenge

Bacterial strain *A. sobria* is from the collection of our laboratory, which was isolated from a diseased fish. A single colony was picked out from an agar plate and grown in LB medium (028324 and 028334, Huankai Microbial Co., Ltd., Guangzhou, P.R. China) overnight (16 h) up to OD_600_ value 2.0. The overnight culture was diluted into fresh LB medium at 1:100 and grown with shaking at 200 rpm at 30°C until the OD_600_ of the culture reached 1.0. Followed by centrifugation at 10,000 rpm for 10 min, bacterial cells were pelleted. After being washed with 0.85% saline solution three times, these bacterial cells were suspended in saline solution and used for bacterial challenge. Each tilapia was intraperitoneally injected with 10 µl of 1 × 10^5^ CFU *A. sobria* (LD_50_) using a microsyringe (F519160, Sangon Biotech Co., Ltd., Shanghai, China) as an experiment group and was intraperitoneally injected with the same volume of saline solution as a control (n = 30 for each treatment). The infective dose of *A. sobria* used was previously determined at 33°C. These tilapias were observed for symptoms twice daily for 15 days for accumulative death. The symptom of infected fish includes slight bleeding around the eyes, spotty bleeding around the gill cover, inflated intestinal tract, and congestive necrosis spleen, suggesting that these tilapias died of multiple-organ hemorrhagic septicemia (30, 31). Germs were isolated for identification of 16S rDNA to quarantine that the infection was caused by *A. sobria*.

### Gas Chromatography–Mass Spectrometry Analysis

To study the metabolic response of tilapia under different temperatures, metabolite abundance was measured by a GC-MS platform. Sample preparation was carried out as previously described (27). In brief, these challenged tilapias and control were euthanized in ice slush for at least 10 min following cessation of gill movement. The fish were rinsed with distilled water and then wiped thoroughly. Spleens were removed ascetically, where 25 mg of spleen was cut. Three spleens were mixed for one sample and then immediately immersed in 1 ml cold methanol. The samples were sonicated for 5 min at a 10-W-power setting using the Ultrasonic Processor (JY92-IIDN, Scientz, China), followed by centrifugation at 12,000 rpm in 4°C for 10 min. Supernatant was collected, and 10 µl ribitol (0.1 mg per mL, Sigma-Aldrich, USA) was added into each sample tube as an internal quantitative standard. The supernatant was concentrated for metabolite derivatization of GC-MS analysis. Every experiment was repeated by five biological replicates. GC-MS detection and spectral processing for GC-MS were carried out using the Agilent 7890A GC equipped with an Agilent 5975C VL MSD detector (Agilent Technologies, USA) as previously described (27).

### Exogenous Administration of Myo-Inositol and Bacterial Challenge

To investigate the effect of exogenous *myo*-inositol on resistance to infection, tilapias (n = 120) were randomly divided into four groups, including three treatment groups (n = 30 per group) and one control group (n = 30), and acclimatized for seven days at 33°C. For the three treatment groups, tilapias were intraperitoneally injected with 200, 400, or 800 µg myo-inositol per fish, where 400 µg myo-inositol was calculated by the increased myo-inositol/g spleen at 33°C × fish weight; n = 30 for each dose (Sigma-Aldrich, USA). For the control group, tilapias were injected with saline only (n = 30). The injection was conducted once daily for 3 days. Finally, tilapias were challenged by intraperitoneal injection of *A. sobria* with 10 µl of 1 × 10^5^ CFU and fish mortality was observed for 15 days for accumulative death. The bacterial challenge was performed twice.

### Measurement of Enzyme Activity

To investigate the effect of exogenous *myo*-inositol on metabolic flux, enzyme activity was measured as previously described (27). In brief, spleens were removed from three tilapias, rinsed with precooled 1× PBS (50 mM, pH 7.4; Invitrogen, USA), resuspended in lysate buffer, and disrupted by sonication for 5 min at a 10-W-power setting. Supernatant with 100 and 250 µg was applied for pyruvate dehydrogenase (PDH) and α-ketoglutarate dehydrogenase (KGDH) assay and succinate dehydrogenase (SDH) and malate dehydrogenase (MDH) assay, respectively. The enzymatic assay was carried out in a 96-well plate with a final volume of 200 µl by mixing an equal volume of the protein sample and reaction buffer.

### RNA Isolation and qRT-PCR

To investigate the expression of innate immunity genes, the same procedures of the exogenous administration of *myo*-inositol and bacterial challenge as earlier described were performed to collect spleens, an important immune organ, for RNA isolation at 0, 3, 6, and 9 h postinfection of 1 × 10^3^ CFU *A. sobria*. One spleen was used as a biological sample. Each experimental group contained six biological samples, which were subjected to two technical replicates, respectively. qRT-PCR was performed as previously described (32). In brief, primers for each gene are listed in **Table 1** and each primer pair was specific. Actin, tubulin, and GAPDH genes were chosen as the internal control. The relative expression of each gene was determined by the comparative threshold cycle method (2*^-ΔΔCT^* method).

**Table 1:** Primers used for qRT-PCR analysis.

Gene	Accession number	Primer	Sequence (5′–3′)
*actin*	NC_031969	Forward	AATCGTGCGTGACATCAAAG
		Reverse	GACGTCGCACTTCATGATG
*gapdh*	NM_001279552	Forward	TTA AGG AAG CCG TCA AGA AG
		Reverse	CAG CAC CAG CAT CAA AGA
*ef1α*	NM_001279647	Forward	CTACGTGACCATCATTGATGCC
		Reverse	AACACCAGCAGCAACGATCA
*il-1β*	NC_031977	Forward	GCGTGCCAACAGTGAGAA
		Reverse	CAGGAGGGACGGAAGGGAT
*il-6*	NC_031972	Forward	ATGCCTGGCGTTGAGTACCT
		Reverse	CAAAATCGCTGACGTGATTGA
*il-8*	NC_031971	Forward	GTATGCCGCCAATCAGCC
		Reverse	GCTCCGTTTGCCAGTCCAG
*il-10*	NC_031970	Forward	CCAATCAGCCGTGACTACAACA
		Reverse	GTGGAATGAGGGTTCAGACAAA
*il-21*	NC_031966	Forward	TCGGGAGACTCTGCTTGAACA
		Reverse	TGTGAGTGGCAGGTTGAGCAG
*tnf-α*	NC_031985	Forward	GTCGTCGTGGCTCTTTGTTTAG
		Reverse	GCCTTGGCTTTGCTGCTGAT
*inf-γ*	NC_031981	Forward	GCATCTGCCAATGTCTTCACAC
		Reverse	GCTGCTGTTCTTGCCTTTACTG
*tlr-1*	NC_031981	Forward	GAGACCGGACTGCACGGCTAT
		Reverse	ACTCAGTTCCTTCCAGCGTTT
*cox2*	NC_013663	Forward	GGGGATTCAACTGGAACTACTATTC
		Reverse	AAGATCTCGGCTTCGATTCTTAT
*lysozyme C*	NC_031967	Forward	CAGATAAATAGCCGCTGGTGG
		Reverse	GCTGTGATGCCTTGTTCCCTA

### Bioinformatics Analyses

Data transformations and manipulations were done using Excel. The Mann–Whitney U test (α = 0.05) with SPSS 23.0 (IBM, USA) was used to compare the difference in abundance of metabolites between two groups. The MetaboAnalyst online website (www.metaboanalyst.ca) was adopted to perform a multivariate statistical analysis of the metabolomic data (33, 34). Z-score analysis was carried out using the following formula: $Z=\frac{x_{ij}-AVGi}{SD_{i}},$ x_ij_ represented metabolites’ peak area, AVG_i_ represented the average of the control group, and SD_i_ represented the standard deviation of the control group. Enriched metabolic pathways were identified using the MetaboAnalyst online website (www.metaboanalyst.ca) (5, 34). Prism v5.01 (GraphPad, La Jolla, CA, USA) was used to draw the histogram and the scatter plot. Comparative metabolic pathway analysis between the two groups was performed using iPath 2.0 (https://pathways.embl.de/) (35).

For the data processing of qRT-PCR and enzyme activity, non-parametric Kruskal–Wallis one-way analysis with Dunn’s multiple-comparison *post hoc* test was used using SPSS 23.0; p < 0.05 and p < 0.01 were considered significant.

## Results

### Tilapias Show Distinct Susceptibility to *A. sobria* at Different Temperatures

**Figure 1:**
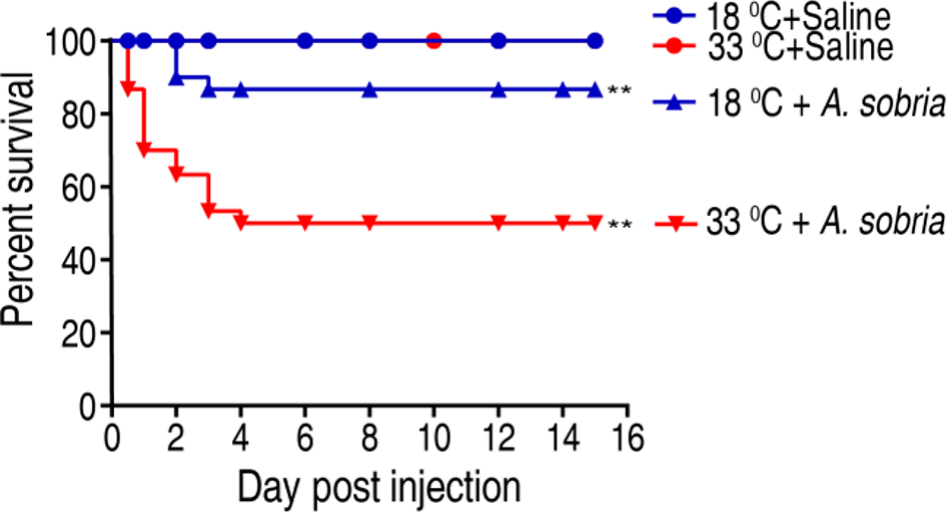
Survival of GIFT (genetically improved farmed tilapia, *Oreochromis niloticus*) to *A. sobria* infection cultured at 18°C and 33°C. Tilapias (n = 30 per group) were acclimated at 18°C and 33°C for 7 days before bacterial challenge. For bacterial infection, tilapias were injected with 10 µl 1 × 10^5^ CFU/fish *A. sobria* or 10 µl saline solution as negative control. Accumulative death was monitored for a total of 15 days. **p < 0.01.

To investigate the effect of water temperature on tilapias’ ability against bacterial infection, tilapia were cultured at 18°C or 33°C and challenged with 1 × 10^5^ CFU of *A. sobria*, which is an LD_50_ dose obtained in pretest. Survival rates were different between the two temperatures as assessed by cumulative survival rates until 15 days postinfection. The survival rate of the fish was 86.67% and 50% at 18°C and 33°C, respectively (**Figure 1**). These results indicate that tilapias are more susceptible to *A. sobria* infection at 33°C than at 18°C.

### Metabolic Profile of Tilapia Reared at 18°C and 33°C

**Figure 2:**
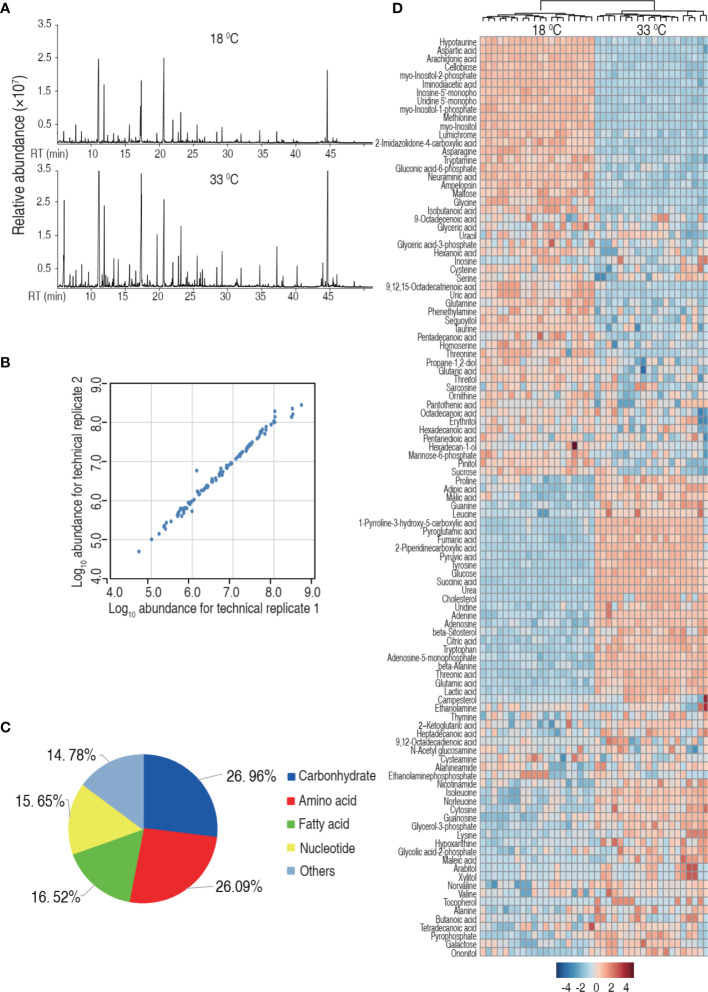
*Tilapia* cultured at 18°C or 33°C had a different metabolome. **(A)** Representative total ion current chromatogram from 18°C or 33°C of samples. **(B)** Reproducibility of the metabolomic profiling platform used in the discovery phase. Abundance of metabolites quantified in samples over two technical replicates is shown. The Pearson correlation coefficient between technical replicates varies between 0.9307 and 0.9996. **(C)** Categories of the differential metabolites. One hundred fifteen metabolites with differential abundance are searched against in KEGG for their categories, and the pie chart is generated in Excel 2010 (Microsoft, USA). **(D)** Heat map of unsupervised hierarchical clustering of different metabolites (row). Blue and red indicate decrease and increase of the metabolites scaled to mean and standard deviation of row metabolite level, respectively (see color scale).

Then, we investigated the effect of water temperature on tilapia metabolome. Spleens were surgically removed from tilapia that was maintained at 18°C and 33°C for a week. The spleens were homogenated and prepared for GC-MS-based metabolomic analysis. Ten individuals with two technical repeats were carried out in each group, yielding a total of 40 data sets. Representative total ion current chromatograms from the 18°C and 33°C groups are listed in **Figure 2A**. A total of 280 aligned individual peaks were obtained from each sample. The correlation coefficients between technical replicates varied between 0.9307 and 0.9996, assuring the reproducibility of the data (**Figure 2B**). A total of 115 metabolites were identified, after the removal of the internal standard and any known artificial peaks. According to the annotation of KEGG (http://www.kegg.jp/) and NCBI PubChem (https://pubchem.ncbi.nlm.nih.gov/), the metabolites were classified into five categories, carbohydrate (26.96%), amino acid (20.06%), nucleotide (15.65%), fatty acid (16.52%), and others (14.78%) (**Figure 2C**). Metabolomic profiles of the two groups were displayed as a heat map (**Figure 2D**). These results indicate that the tilapia have differential metabolism when cultured at different temperatures.

### Differential Metabolome Related to Higher Water Temperature

**Figure 3:**
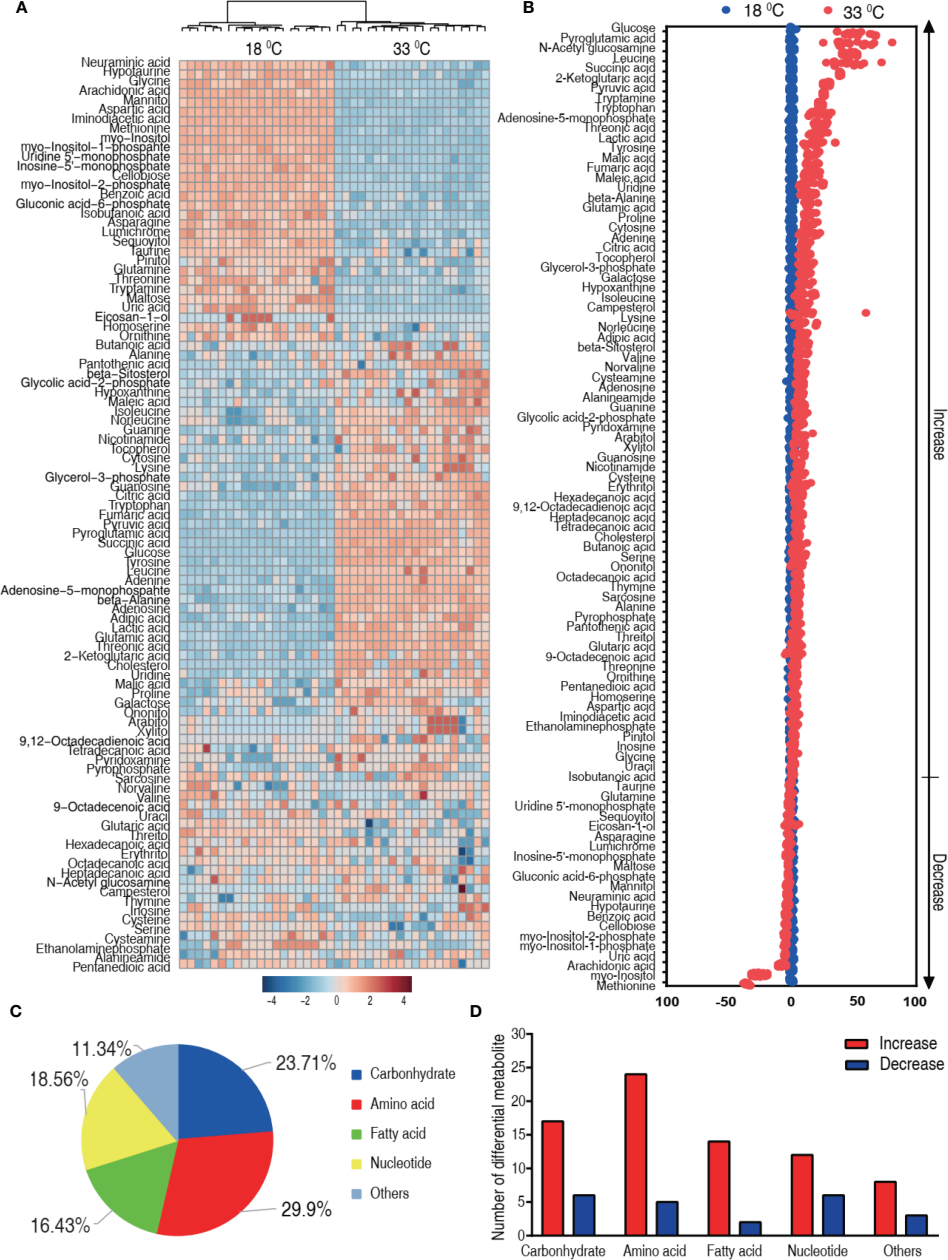
Differential metabolic profiles of tilapias cultured at different temperatures. **(A)** Heat map showing 97 differential metabolites. Red and blue indicate increase and decrease of metabolites relative to the median metabolite level, respectively (see color scale). **(B)** Z-score plot of differential metabolites based on the 18°C group. Each point represents one metabolite in one technical repeat and colored by sample types. **(C)** The different metabolites were classified into five categories, carbohydrate (23.71%), amino acid (29.9%), nucleotide (16.43%), fatty acid (18.56%), and others (11.34%). **(D)** Number of increased and decreased metabolites in these categories **(C)**, 17 and 6 (carbohydrate), 24 and 5 (amino acid), 14 and 2 (nucleotide), 12 and 6 (fatty acid), and 8 and 3 (others), respectively.

We speculated that the difference in the mortality was related to the characteristic metabolome constructed by differential metabolites. Thus, the differential metabolome that distinguished 33°C groups from 18°C was explored. A two-sided Mann–Whitney U test coupled with permutation test was used to identify the differential abundance of metabolites between the 18°C group and the 33°C group. A total of 97 differential abundance of metabolites (*p* < 0.01) was identified at the 33°C group, which corresponded to a false discovery rate (FDR) less than 0.048737. The identified metabolites are shown in **Figure 3A** as a heat map, where the two groups were clearly separated. The Z-score plot spans from 52.52 to -214.06 in these groups. In comparison to the 18°C control group, 75 metabolites were increased and 22 metabolites were decreased in the 33°C group (**Figure 3B**). Among them, *myo*-inositol was the most depressed metabolite. The differential metabolites were classified into five categories, carbohydrate (23.71%), amino acid (29.9%), nucleotide (16.43%), fatty acid (18.56%), and others (11.34%) (**Figure 3C**). Increased and decreased metabolites in these categories are shown in **Figure 3D**, showing that the number of increased metabolites is higher than that of decreased metabolites. These results indicate that the metabolome at 33°C is different from the metabolome at 18°C.

### Crucial Biomarkers Related to Higher Water Temperature

**Figure 4:**
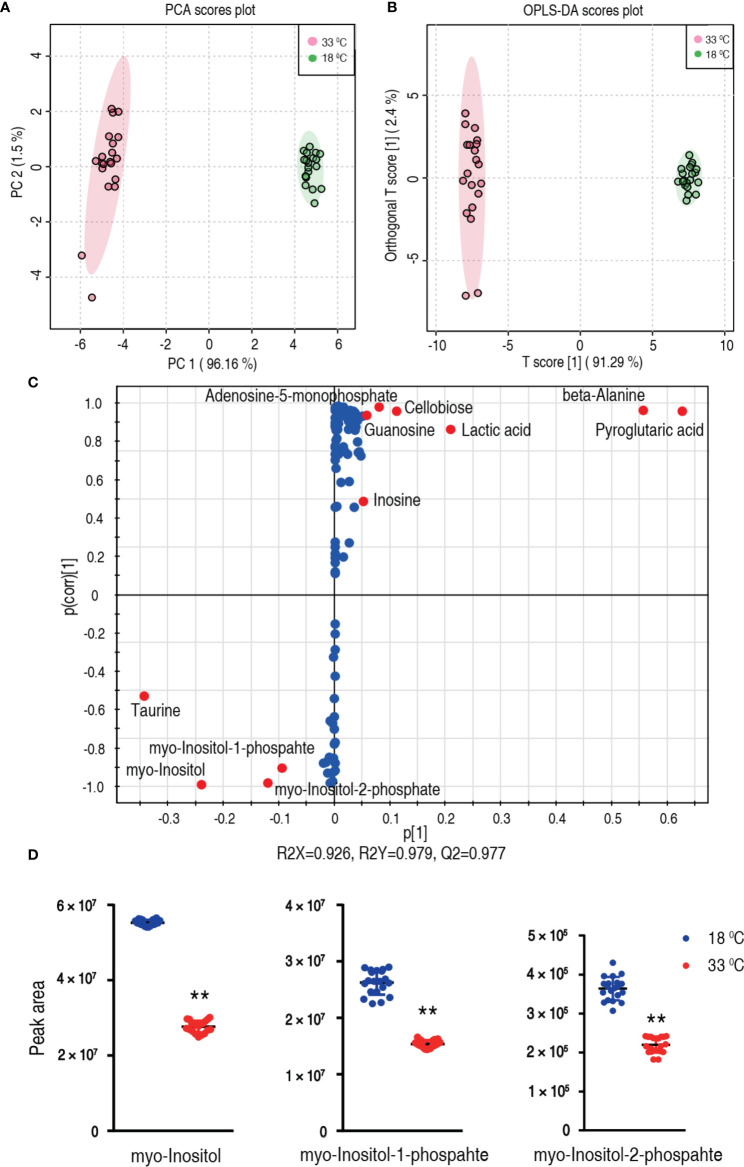
Identification of crucial metabolites. **(A)** PCA analysis of 18°C and 33°C groups according to the treatment set. Each dot represents the technological replicate analysis of samples in the plot. PC 1 and PC 2 used in this plot explain 97.66% of the total variance which allows confident interpretation of the variation. **(B)** OPLS-DA analysis of 18°C and 33°C groups. Dots represent technological replicates. Component 1 (T score [1] = 91.29%) and Component 2 (orthogonal T score [1] = 2.4%) of OPLS-DA explain 93.69% of the total variance. **(C)** S-plot generates from OPLS-DA (R2X = 0.926, R2Y = 0.979, Q2 = 0.977). Predictive component p[1] and correlation p(corr)[1] differentiate 18°C from 33°C. Dot represents metabolites, and candidate biomarkers are highlighted in red. **(D)** Scatter plot of *myo*-inositol, myo-inositol-1-phosphate, and myo-inositol-2-phosphate. **p < 0.01.

To explore the most crucial metabolites differentiating 33°C groups from 18°C groups, principal component analysis (PCA) and orthogonal partial least square discriminant analysis (OPLS-DA) were conducted to recognize the sample pattern. PC1 (96.16%) and PC2 (1.5%) of PCA (**Figure 4A**) and Component 1 (T score [1] = 91.29%) and Component 2 (orthogonal T score [1] = 2.4%) of OPLS-DA (**Figure 4B**) separated the samples into two quarters. Using unsupervised pattern recognition and supervised pattern recognition, PC 1 and Component 1 clearly separate the 33°C group from the 18°C group. Discriminating variables were shown with the S-plot (**Figure 4C**) (R^2^X = 0.926, R^2^Y = 0.979, Q^2^ = 0.977) when cutoff values were set as greater or equal to 0.05 and 0.5 for the absolute values of covariance p and correlation p(corr), respectively. Eleven biomarkers screened by component p[1] and p(corr)[1] are shown in **Figure 4C** in red oval. Among these biomarkers, the abundance of five biomarkers was reduced including *myo*-inositol and its derivatized forms, myo-inositol-1-phosphate and myo-inositol-2-phosphate. The abundance of these metabolites is shown in **Figure 4D**. These results with the above analysis on the most depressed myo-inositol indicate that myo-inositol can be identified as a crucial biomarker.

### Pathway Enrichment Related to Higher Water Temperature

**Figure 5:**
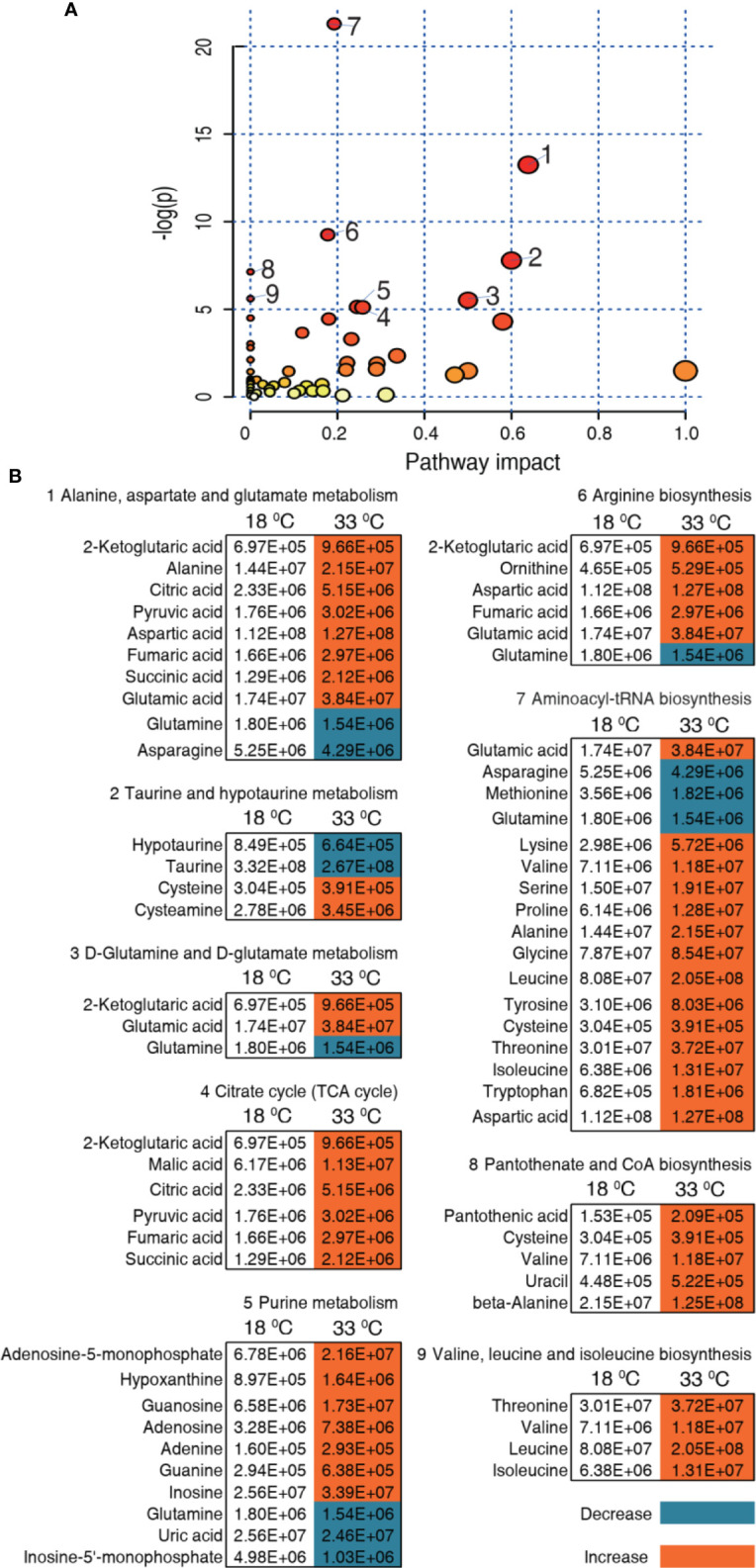
Pathway enrichment and analysis. **(A)** Pathway enrichment of differential metabolites in the 33°C group compared with the 18°C group. In terms of impact value from the largest to the smallest, 1 to 9, respectively, represent alanine, aspartate and glutamate metabolism; taurine and hypotaurine metabolism; D-glutamine and D-glutamate metabolism; citrate cycle (TCA cycle); purine metabolism; arginine biosynthesis; aminoacyl-tRNA biosynthesis; pantothenate and CoA biosynthesis; and valine, leucine, and isoleucine biosynthesis. Significantly enriched pathways are selected to plot. **(B)** Integrative analysis of metabolites in significantly enriched pathways. Red and blue indicate increased and decreased metabolites, respectively.

We further investigated the pathways that were impacted in these two temperatures, which are important indicators for the alterations of the metabolome. Using MetaboAnalyst 4.0, 9 metabolic pathways were enriched for the 33°C group (**Figure 5A**). According to impact value, the nine most impacted pathways included alanine, aspartate, and glutamate metabolism; taurine and hypotaurine metabolism; D-glutamine and D-glutamate metabolism; citrate cycle (TCA cycle); purine metabolism; arginine biosynthesis; aminoacyl-tRNA biosynthesis; pantothenate and CoA biosynthesis; and valine, leucine, and isoleucine biosynthesis. Integrative analysis of metabolites in the enriched pathways is carried out, where red and blue indicate increased and decreased metabolites, respectively. Of particular interest is that all metabolites of the TCA cycle were increased in the 33°C group (**Figure 5B**). These results indicate that the increased TCA cycle forms a characteristic feature in tilapias that survived at 33°C.

**Figure 6:**
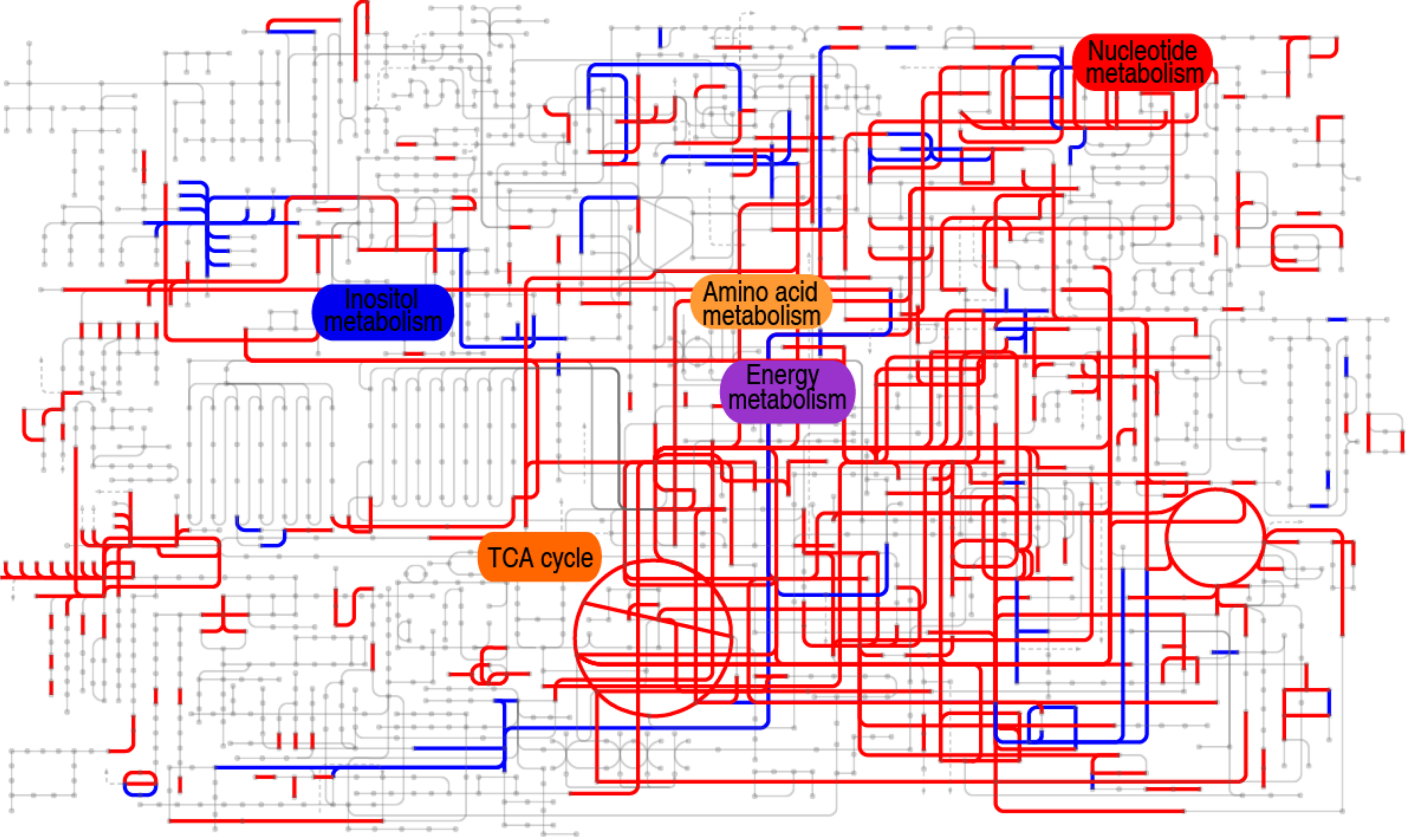
Comparative metabolic pathway analysis between the 18°C and 33°C groups. Analysis of the metabolic profiles resulting from tilapias cultured at 18°C and 33°C provides a better insight into the effect of 97 differential abundances of metabolites (*p* < 0.01). Based on the KEGG compound (http://www.kegg.jp/kegg/compound/), metabolic network pathways in tilapias are further analyzed with iPath2.0 (http://pathways.embl.de/iPath2.cgi). Red line represents increase in the 33°C group; blue line represents decrease in the 33°C group.

Furthermore, comparative metabolic pathway analysis between the 18°C and 33°C groups was performed in iPath 2.0. The iPath analysis generates a global view and provides a better insight into the effects of culture temperature on the metabolic profile of the tilapias, where red line represents increased pathways in the 33°C group and blue line represents decreased pathways in the 33°C group. We found activation of the TCA cycle, amino acid metabolism, energy metabolism, and nucleotide metabolism as the elevated metabolic pathways and inactivation of carbohydrate metabolism and weak inositol metabolism as the reduced production of key metabolites (**Figure 6**). The integrated analysis of the metabolites with differential abundance and pathway enrichment suggests that the reduction of *myo*-inositol abundance and the activation of the TCA cycle play a role in fish death at higher water temperature.

### Exogenous *Myo*-Inositol Elevates Survival of the Infected Tilapia at 33°C *via* Inhibiting the TCA Cycle

**Figure 7:**
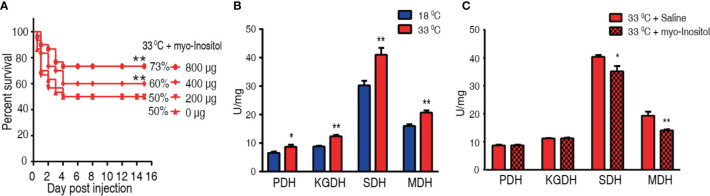
*Myo*-inositol impacts the TCA cycle and promote tilapia’s ability against bacterial infection. **(A)** Survival of tilapias post *A sobria* infection in the presence of *myo*-inositol. Tilapias were treated with saline control (0 µg per fish) or different doses of *myo*-inositol (200, 400, and 800 µg per fish) at 33°C for 3 days, followed by bacterial challenge through intraperitoneal injection (1 × 10^5^ CFU *A. sobria*). The accumulative fish death was monitored for a total of 15 days postinfection (n =30 per group). **(B)** Activity of PDH, KGDH, SDH, and MDH of spleens at 18°C and 33°C. **(C)** Activity of PDH, KGDH, SDH, and MDH of spleens in the presence or absence of 800 µg *myo*-inositol per fish. Values are means ± SEM (n = 6 per group), and statistic difference is analyzed with non-parametric Kruskal–Wallis one-way analysis with Dunn’s multiple-comparison *post hoc* test. *p < 0.05; **p < 0.01 **(B, C)**.

We hypothesized that elevation of *myo*-inositol in tilapia may promote fish survival to bacterial challenge at 33°C. To explore this idea, tilapias were intraperitoneally injected with 200, 400, or 800 µg *myo*-inositol per fish, followed by the challenge with *A. sobria* at 33°C. The different doses of exogenous *myo*-inositol showed a protective impact on the bacterial infection in a dose-dependent manner (**Figure 7A**). The occurrence may be related to the possibility that exogenous *myo*-inositol impacts the TCA cycle since the elevated TCA cycle is associated with the susceptibility at 33°C. Activity of PDH, SDH, KGDH, and MDH in the TCA cycle was quantified in tilapias cultured at 18°C and 33°C. Higher activity of the four enzymes was detected at 33°C than at 18°C. In detail, PDH activity was increased from 6.55 ± 0.48 U/mg at 18°C to 8.67 ± 0.75 U/mg at 33°C; KGDH activity was increased from 8.83 ± 0.28 U/mg at 18°C to 12.39 ± 0.44 U/mg at 33°C; SDH activity was increased from 30.28 ± 1.59 U/mg at 18°C to 41.04 ± 2.39 U/mg at 33°C; MDH activity was increased from 16.03 ± 0.59 U/mg at 18°C to 20.73 ± 0.75 U/mg at 33°C) (**Figure 7B**). Then, the activity was further detected at 33°C in the absence or presence of 800 µg exogenous *myo*-inositol. The exogenous *myo*-inositol inhibited the activity of SDH and MDH but did not affect the activity of PDH and KGDH. Specifically, SDH activity was reduced from 42.35 ± 0.66 to 36.89 ± 2.00 U/mg and MDH activity was reduced from 20.27 ± 1.56 to 14.79 ± 0.34 U/mg at 33°C compared with that at 18°C (**Figure 7C**). These results indicate that *myo*-inositol reprograms the TCA cycle, leading to the elevated survival in *A. sobria* infection at 33°C.

### Expression of Innate Immune Genes Modulated by Myo-Inositol

**Figure 8:**
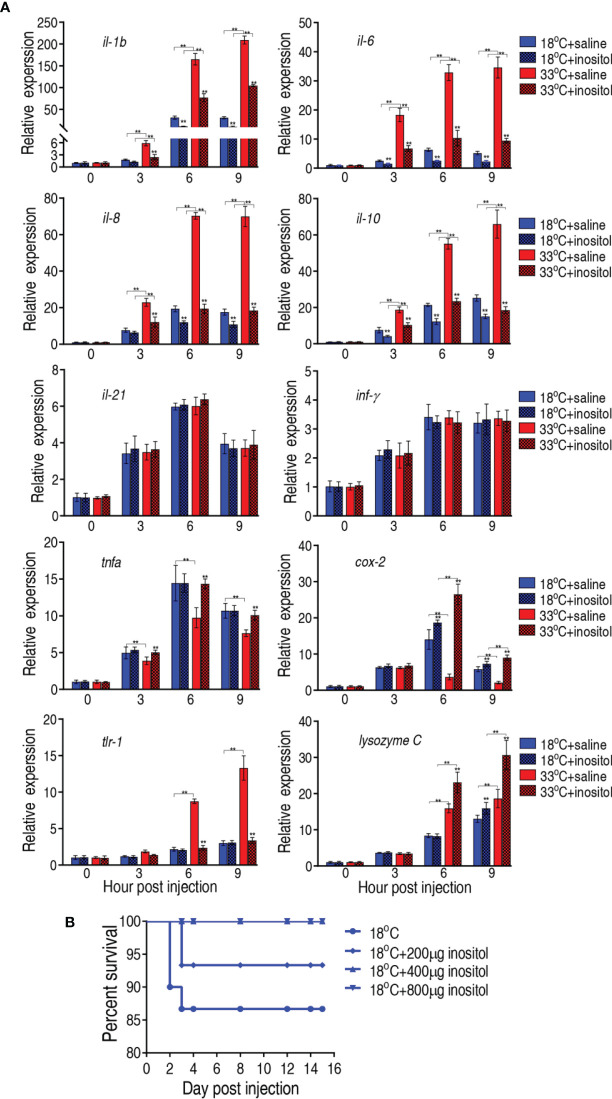
Innate immune responses of tilapias. **(A)** qRT-PCR for innate immune genes of tilapia treated with saline solution (control) or 800 µg *myo*-inositol for 3 days following *A. sobria* challenge through intraperitoneal injection (1 × 10^3^ CFU *A. sobria*). Spleens were collected at 0, 3, 6, and 9 h postinjection for RNA extraction and qRT-PCR. Values are means ± SEM from six biological replicates. **p < 0.01. **(B)** Survival of tilapias post *A. sobria* infection in the presence of *myo*-inositol. Tilapias were treated with saline control (0 µg per fish) or different doses of *myo*-inositol (200, 400, and 800 µg per fish) at 18°C for 3 days, followed by bacterial challenge through intraperitoneal injection (1 × 10^5^ CFU *A. sobria*). The accumulative fish death was monitored for a total of 15 days postinfection (n =30 per group).

Elimination of bacterial pathogens is dependent on immunity. It is reasonable that *myo*-inositol is related to immune response in the temperature-mediated survival. To demonstrate this, we quantified the dynamic transcriptional level of 10 innate immune genes, *il-1b*, *il-6*, *il-8*, *il-10*, *il-21*, *tnf-α*, *inf-γ*, *tlr-1*, *cox 2*, and *lysozyme C*, at 0, 3, 6, and 9 h postinfection after a 3-day injection of *myo*-inositol. The expression of the genes represents four patterns. The expression of *il-1b*, *il-6*, *il-8*, and *il-10* was increased along with the infection time, where the expression of the four genes was higher at 33°C than at 18°C. However, *myo*-inositol attenuated the expression at both groups. The expression of *il-21* and *inf-γ* represents the second pattern, where the expression of both genes was increased upon infection but was unaffected by inositol. In the third pattern, the expression of *tnf-α* and *cox 2* was increased and reduced, respectively, during infection, and interestingly, inositol dramatically increased the gene expression at 33°C but only slightly increased at the 18°C group. In the fourth pattern, the expression of *tlr-1* and *lysozyme C* was elevated with time postinfection, where a higher expression was detected at 33°C than at 18°C but not regulated by *myo*-inositol (*tlr-1*) or regulated by *myo*-inositol (*lysozyme C*) (**Figure 8A**). These results indicate that *myo*-inositol modulates innate immune response that is related to the survival ability against *A. sobria* infection at 33°C. To further explore whether the *myo*-inositol regulation for bacterial infection is effective at 18°C for understanding the role of the TCA cycle in tilapias against the bacterial infection, the same experiment on bacterial challenge was carried out at 18°C. Fish survival was elevated in a *myo*-inositol dose-dependent manner at 18°C (**Figure 8B**) in comparison with the event that the elevation of inflammatory cytokines *il-1b*, *il-6*, *il-8*, and *il-10* was attenuated by exogenous *myo*-inositol, as described above (**Figure 8A**). The finding supports the conclusion that the TCA cycle is related to survival of tilapias against *A. sobria* infection. These results indicate that *myo*-inositol regulates innate immune response which contributes to the survival ability against *A. sobria* infection.

## Discussion

Water temperature affecting tilapia’s ability against bacterial infection is documented (8, 10, 11). However, the underlying mechanisms are largely unknown. The present study explores the metabolic mechanisms accounting for such capability. Tilapias can grow between 16°C and 38°C, with an optimum range between 25°C and 28°C (36). For identifying big contrast biomarkers between higher and lower water temperatures, 33°C and 18°C were selected. It is clear that tilapias have differential metabolomes between 18°C and 33°C, which are related to higher and lower survival after *A. sobria* infection, respectively. The lower water temperature metabolome is more efficient against bacterial infection than the higher water temperature metabolome. This is because the metabolome is associated with host anti-infective innate immunity (11, 13, 37). Thus, metabolome reflects the metabolic state that demonstrates host ability against bacterial pathogens. Notably, low temperature inhibits bacterial proliferation, which affects bacterial virulence and thereby leads to fish higher survival. However, exogenous *myo*-inositol promoted the survival of fish challenged by *A. sobria* at 33°C than at 18°C, indicating metabolic modulation plays a role in the temperature-related survival.

In the present study, the increased TCA cycle and the decreased level of *myo*-inositol were identified as the metabolic signature to differentiate the higher water temperature metabolome from the lower water temperature metabolome. More importantly, there is a close link between the TCA and *myo*-inositol that *myo*-inositol inhibits the activity of SDH and MDH and thereby weakens the TCA cycle. This is a previously unrecognized correlation of the events that can explain that the reduced level of *myo*-inositol is accompanied with the increased TCA cycle. Since the elevated TCA cycle is related to the low survival of tilapia at 33°C, the reduced TCA cycle mediated by *myo*-inositol contributes to the survival of the fish in the same water temperature. Meanwhile, *myo*-inositol also potentiates fish against the infection at 18°C. Thus, *myo*-inositol can be a potential drug to elevate the survival of tilapia at the higher water temperature. These findings indicate that the elevation of the TCA cycle is a stress response at higher water temperature. However, the response affects the survival of tilapia. Recently, the relationship between the TCA cycle and bacterial infection has been explored. In response to zebrafish infection caused by *Vibrio alginolyticus*, the activation and inactivation of the TCA cycle are responsible for the survival and death, respectively. Exogenous malate boosts the TCA cycle to regulate the expression of innate immunity genes *via* taurine and thereby enhances the survival of zebrafish to the *V. alginolyticus* infection (20, 27). However, exogenous glucose enhances tilapia against *Edwardsiella tarda* infection in association with the attenuation of the TCA cycle (24). Therefore, the role of the TCA in bacterial infection can be an ideal breakthrough point to understand the metabolic modulation strategy to the infection.

The present study further showed that differential innate immune responses were detected between 18°C and 33°C, which were consistent with the differential metabolomes between the two temperatures. These findings motivated us to hypothesize that the innate immune response is regulated by a crucial metabolite identified from the differential metabolomes. Therefore, the effect of *myo*-inositol on the expression of innate immune genes was explored. Exogenous *myo*-inositol modulated the expression of the most measured genes. These results indicate that the metabolic state is closely related to the innate immune state and a crucial metabolite can restore the host’s anti-infective innate immune response (30, 38–40).

A line of studies has indicated that *myo*-inositol is crucial in several metabolic and regulatory processes, such as second messenger, lipid signaling, osmolarity, immunomodulation, glucose, and insulin metabolism (32, 41–44). Exogenous *myo*-inositol appears to be a valuable alternative for treatment of several diseases as well as for improvements in metabolic performance (29, 45, 46). However, information regarding action of *myo*-inositol against bacterial infection at the higher water temperature is not defined. The present study reveals the previously unknown function of *myo*-inositol in elevating anti-infective ability at the higher temperature. Recently, our group has shown that *myo*-inositol improves the host’s ability to eliminate balofloxacin-resistant *Escherichia coli*, which is related to promotion of the phosphorylation of Akt1 (47). Other authors indicate that *myo*-inositol is involved in signaling the immune response through phosphorylation (41, 42). The present study identified that not only *myo*-inositol but also myo-inositol-1-phosphate and myo-inositol-2-phosphate were also significantly reduced at higher water temperature. Therefore, the phosphorylation of *myo*-inositol is a clue to further understand the mechanisms by which *myo*-inositol restores the ability against bacterial infection at the higher temperature.

The present study investigated 10 innate immune genes, which are categorized to β-trefoil cytokines (*il*-*1b*), type I α helical cytokines (*il*-*6*, *il*-*10*), chemokine (*il*-8), TLR family (*tlr 1*), cyclooxygenase 2 (*cox 2*), and β-jellyroll cytokines (*tnf-α*) (48). Among the 10 innate immune genes detected, the expression was elevated with time postinfection at 18°C and 33°C. However, higher *il-1b*, *il-6*, *il-8*, *il-10*, and and *lysozyme C* and lower *cox 2* were measured at 33°C than at 18°C, where *il-1b*, *il-6*, *il-8*, and *il-10* levels were attenuated and *cox 2* and *lysozyme C* levels were increased by exogenous *myo*-inositol. In addition, the elevated *tlr-1* was reverted by exogenous *myo*-inositol. These results are acccompanied with the increased survival of tilapias infected by *A. sobria* and treated by *myo*-inositol at 33°C and 18°C, suggesting that *myo*-inositol regulation to innate immune response is related to the tilapia survival that is associated with water temperature. In addition, juvenile tilapias were used in the present study. This is because juvenile tilapias with an immature immune system are extremely vulnerable to infection, which causes huge economic losses and thereby requires to be investigated for solution (49–51). Our finding on exogenous myo-inositol modulation to elevate survival provides an effective approach to cope with bacterial infection in juvenile tilapias.

In summary, the present study explores the relationship among water temperature, metabolic state, and innate immune response against bacterial infection. Our results showed that lower survival of tilapia infected with *A. sobria* at 33°C than 18°C is related to a respective metabolome and innate immune response. Furthermore, there is a close link between the metabolome and the innate immune response, i.e., higher-temperature metabolome and lower-temperature metabolome decide strong and weak innate immune responses, respectively. Interestingly, exogenous *myo*-inositol restores the reasonable innate immune response against bacterial infection at the higher and lower water temperature. These findings highlight the way in eliminating bacterial pathogens by metabolic modulation.

## Data Availability Statement

The raw data supporting the conclusions of this article will be made available by the authors, without undue reservation.

## Ethics Statement

The animal study was reviewed and approved by the Institutional Animal Care and Use Committee of Sun Yat-sen University. Written informed consent was obtained from the owners for the participation of their animals in this study.

## Author Contributions

HL conceptualized and designed the project. M-JY and MJ performed the experiments and data analysis. HL and X-XP interpreted the data. HL wrote the manuscript. All the authors reviewed the manuscript. All authors contributed to the article and approved the submitted version.

## Funding

This work was sponsored by grants from the Guangzhou Science and Technology Project (201904020042), the Fellowship of China Postdoctoral Science Foundation (2020M683023, 2020TQ0368), the International Exchanges Scheme (NSFC-RS) (319115301830), and the Innovation Group Project of Southern Marine Science and Engineering Guangdong Laboratory (Zhuhai) (311021006).

## Conflict of Interest

The authors declare that the research was conducted in the absence of any commercial or financial relationships that could be construed as a potential conflict of interest.

## Publisher’s Note

All claims expressed in this article are solely those of the authors and do not necessarily represent those of their affiliated organizations, or those of the publisher, the editors and the reviewers. Any product that may be evaluated in this article, or claim that may be made by its manufacturer, is not guaranteed or endorsed by the publisher.
